# An Organizational-Level Intervention to Enhance Sustainable Employability of Long-Term Care Workers: A Process Evaluation of the Healthy Working Approach

**DOI:** 10.1097/JOM.0000000000003419

**Published:** 2025-04-18

**Authors:** Marieke van Kuppenveld, Ceciel H. Heijkants, Madelon L.M. van Hooff, Lander J.G. Vermeerbergen, Cécile R. L. Boot

**Affiliations:** From the Radboud University, Behavioural Science Institute, Nijmegen, The Netherlands (M.v.K., C.H.H., C.R.L.B.); Open Universiteit, Faculty of Psychology, Heerlen, The Netherlands (M.L.M.v.H.); Radboud University, Institute for Management Research, Nijmegen, The Netherlands (L.J.G.V.); Amsterdam UMC, VU University, Department of Public and Occupational Health, Amsterdam, The Netherlands (C.R.L.B.); and Amsterdam Public Health research institute, Societal Participation and Health, Amsterdam, The Netherlands (C.R.L.B.)

**Keywords:** organizational-level intervention, participatory approach, sustainable employability, long-term care, process evaluation

## Abstract

**Objectives:**

The aims of the study were to evaluate whether the Healthy Working Approach was implemented as planned and to gain insight into perceived positive outcomes and barriers and facilitators to the implementation.

**Methods:**

We conducted a mixed methods process evaluation on a team-based approach for sustainable employability of long-term care workers. We examined context, recruitment, reach, dose delivered, dose received, fidelity, satisfaction, perceived positive outcomes, and barriers and facilitators.

**Results:**

Reach was 11% whereas dose delivered, dose received, fidelity, and satisfaction were above 60%. Perceived positive outcomes included improved team coherence, faster problem solving, and professional development. Barriers and facilitators included collaboration in working groups, motivation, and scheduling meetings.

**Conclusions:**

For the teams reached, the approach was implemented according to protocol and perceived as positive. Future implementation should focus on increasing reach and dose delivered from working groups to teams.

## Abstract 2

Population aging and poor working conditions threaten the sustainable employability of long-term care workers. To enhance the sustainable employability of these workers, we developed an organizational-level intervention and evaluated the implementation of this intervention via a process evaluation. Our findings support the long-term care sector in realizing a tenable workforce.

## Graphical abstract

**Figure:**
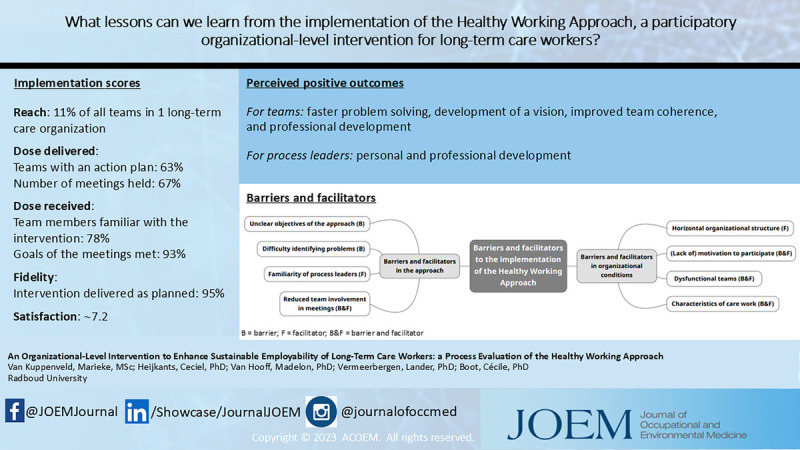

**LEARNING OUTCOMES**

After reading our study, the reader will be able to:

1. Describe barriers and facilitators to the implementation of the Healthy Working Approach and parts of the implementation that were successful and parts that need to be improved.
2. Describe the perceived positive outcomes of this approach for long-term care workers and process leaders.
3. Innovate the participatory approach by including self-managing teams and process leaders from within the organization.

The sustainable employability of long-term care workers is at risk. First, population aging leads to a growing number of older adults in demand for their services and a decrease in the working population, as large cohorts of older workers are approaching the retirement age.^1^ Second, long-term care workers face significant psychosocial hazards related to both the content of their work and the work environment, such as emotionally and physically heavy workloads, demanding work schedules (eg, evening and night shifts), and limited development opportunities.^2,3^ These psychosocial hazards further exacerbate job dissatisfaction and high turnover, resulting in difficulties in the recruitment and retention of workers.^1^ Therefore, it is crucial to focus on the ability of long-term care workers to perform their work with the preservation of health and well-being during their working life, that is, their sustainable employability.^4^

To enhance sustainable employability, interventions have been developed that reduce psychosocial hazards at the workplace. According to a recent umbrella review by Aust and colleagues (2023), organizational-level interventions that focus on reducing psychosocial hazards showed moderate-quality evidence to improve health outcomes.^5^ Additionally, intervention studies using a stepwise participative approach—from problem identification to an action plan aimed at improving process aspects in work content and work environment—were associated with positive outcomes for workers.^5–8^

Drawing on these insights, we developed the Healthy Working Approach. The Healthy Working Approach is an organizational-level intervention that aims to enhance the sustainable employability of long-term care workers in self-managing teams. It involves a participatory approach in which teams prioritize and act upon team-specific barriers.^9^ The focus on the team is important, as it is the primary unit of decision making. Since teams differ in terms of residents, team composition, team culture, and challenges, a one size fits all approach will likely not benefit all teams.^10^ The participatory approach allows teams to select their own barriers and to draw up SMART action plans that best suit their team.

A randomized controlled trial showed that the Healthy Working Approach had a positive significant effect on the satisfaction of the need for relatedness in long-term care workers.^11^ However, the trial found no effects on need for recovery or satisfaction of the need for autonomy and competence.^11^ A process evaluation on the Healthy Working Approach is needed to move beyond the black box of the intervention process, to understand which parts of the intervention protocol were or were not followed and to provide possible explanations for the results of the trial.^12^ Moreover, insights into the perceived positive outcomes of the approach and the barriers and facilitators to the implementation that were perceived by stakeholders are valuable to formulate specific directions for future improvements of the protocol and directions for implementation of the approach in other long-term care organizations or beyond. Therefore, the aims of this process evaluation on the Healthy Working Approach were:

1) to evaluate whether the Healthy Working Approach was implemented as planned; and
2) to gain insight into perceived positive outcomes and barriers and facilitators to the implementation of the Healthy Working Approach in a long-term care organization.

## METHODS

We undertook a mixed methods process evaluation on the Healthy Working Approach. This approach was developed based on a needs assessment^10^ and tested in a randomized controlled trial.^11^ For details regarding the methods and development of the approach, we refer to the protocol of this trial.^9^

### The Healthy Working Approach

The approach comprised of three 1-hour meetings for each team. The three basic psychological needs^13^—autonomy, relatedness, and competence—identified through the needs assessment^10^ with our target group were used to structure the meetings. These meetings aimed to construct an action plan to enhance sustainable employability. Moreover, the meetings were guided by a process leader who was a trained employee from within the long-term care organization. The process leader aimed to maintain a safe and confidential environment in which all opinions are valued equally. Each team selected representatives to join a working group who would attend these meetings. The working group had the task of representing the whole team and therefore had to report back to the team after each meeting. The approach aimed to result in improvements that would benefit the whole team. An implementation plan of the Healthy Working Approach is provided in Supplementary File I (http://links.lww.com/JOM/B925).

#### Meeting 1: Problem Analysis

In the initial meeting, the working group brainstormed about team-related issues concerning autonomy, relatedness, and competence. They prioritized and selected two to three highly impactful problems. The chosen issues were shared with the rest of the team that was not part of the working group.

#### Meeting 2: Solutions and Action Plan (1–2 Weeks After Meeting 1)

In the second meeting, the working group brainstormed about solutions for the agreed-upon problems. The solutions could cover various aspects, such as technical and organizational solutions or changing working conditions, and additional support. These solutions were evaluated based on the criteria simplicity, feasibility, support, practicability, and expected effectiveness. Following this, the working group developed action plans (specifically answering: Who does What When?) for the top solutions for each problem, which were shared with the entire team.

#### Meeting 3: Implementation and Evaluation (1–2 Months After Meeting 2)

During the implementation of the solutions, the working group was able to ask the process leader for support where needed. In the third meeting, the progress of the solutions was reviewed (implemented, not implemented, in progress), and adjustments were considered for improvements to the implementation.

### Study Population

The process evaluation of the Healthy Working Approach was administered in one Dutch mid-large long-term care organization with five sites. The organization has a horizontal structure and practices self-managing care teams. The study population of this process evaluation consisted mainly of women aged between 18 and 54 and comprised five process leaders, two executives (director, location manager), one self-management coach, and eight teams including 31 working group members. An overview of the participating teams is displayed in Supplementary File II (http://links.lww.com/JOM/B926).

### Components of the Process Evaluation

The process evaluation of the Healthy Working Approach was based on the components of the Linnan and Steckler framework for process evaluations.^12^ In the present study, we assessed context, recruitment, reach, dose delivered, dose received, and fidelity. In addition, we investigated satisfaction, perceived positive outcomes, and barriers and facilitators to the implementation of the Healthy Working Approach. Table 1 shows how we operationalized each component.

**TABLE 1:** Operationalization of the Components of the Process Evaluation

Component	Operationalization
Context	Aspects of the larger context that influenced intervention implementation.
Recruitment	Procedures to approach and attract teams, working groups, and process leaders.
Reach	Total: the proportion of teams that participated in the intervention versus all teams in the organization.
	Care teams: the proportion of care teams that participated in the intervention versus all care teams in the organization.
Dose delivered	Teams: the number of action plans made by the working groups.
	Working group: the number of meetings planned.
Dose received	Teams: the percentage of the team members that were familiar with the intervention.
	Working group: the degree to which goals were met per meeting.
Fidelity	Working group: the extent to which the meetings were delivered as planned.
Satisfaction	Teams: satisfaction with the intervention.
	Working group: satisfaction with the meetings.
Perceived positive outcomes	Perceived positive outcomes of the intervention.
Barriers & facilitators to the implementation	Elements that hindered or promoted the implementation of the intervention.

### Data Sources

In Table 2, we provide an overview of the data that were used in this study, including the groups of participants involved, the time points, and the process evaluation components the data contributed to.

**TABLE 2:** Overview of the Data Used in this Study

Data	Participants	Time Points	Contributed to
Team questionnaires (*n* = 54)*	Team members	6 and 9 months after a team finished the meetings	Dose received Satisfaction
Working group questionnaires (*n* = 43)*	Working group members	After each meeting	Dose received Satisfaction
Process leader logs (*n* = 13)†	Process leaders	After each meeting	Dose delivered Fidelity
Interviews (*n* = 13)	Working group members Process leaders Executives Self-management coach	After all teams had finished the meetings	Context Perceived positive outcomes Barriers and facilitators to the implementation
Researcher notes	Not applicable	Continuous	Context Recruitment Reach

*Questionnaires of teams that did not finish all three meetings were excluded, as these teams are not taken into account in calculating dose received and satisfaction.

†Logs of meetings with teams that did not finish all three meetings were included, as these teams *are* taken into account in calculating dose delivered.

*Team questionnaires* after 6 and 9 months were sent digitally to all team members who filled out the baseline questionnaire and participated in the approach (*n* = 56). The baseline questionnaire was part of the randomized controlled trial and was administered before the approach was implemented. Two items of this questionnaire were relevant for this process evaluation. These concerned whether participants were familiar with the Healthy Working Approach (yes or no), and if ‘yes’, how satisfied participants were with the Healthy Working Approach (ranging from 0 very unsatisfied to 10 very satisfied).

*Working group questionnaires* were administered after each meeting of the Healthy Working Approach and were filled out on paper. In these questionnaires, all attending working group members were asked how satisfied they were with the meeting (ranging from 0 very unsatisfied to 10 very satisfied) and whether the goal of the meeting was attained on a five point scale (ranging from achieved to a very large extent to not attained).

*Process leader logs* were used to gain more insight into the attendance rate of the meetings, which bottlenecks, solutions, and actions were chosen, and to monitor which actions succeeded and which ones needed follow-up. The process leader logs had a fixed format and were physically handed over by researcher CH to the process leaders and by the process leaders to CH when they were filled out. CH identified missing information in the logs and requested this information by phone if necessary.

*Interviews* were held with working group members, process leaders, executives, and a self-management coach after all teams had finished the meetings of the Healthy Working Approach. The main aim of these semi-structured interviews was to gain insight into the context, the perceived positive outcomes, and the barriers and facilitators to the implementation of the approach. In total, there were 31 working group members, of which we could not reach three (no work email address known to researcher), and 10 working group members never had meetings. Therefore, 18 working group members were asked to participate in an interview. A total of five working group members participated, with another two people sharing experiences as working group members but were mainly included in the analysis for their primary role as process leader or self-management coach. All five process leaders, all executives, and the self-management coach participated.

*Researcher notes* were used to describe the context, recruitment, and reach of the intervention. For example, the researcher retrieved the number of teams in the organization at the start of the implementation and registered how teams and process leaders were recruited.

### Data Analyses

*Context and recruitment* were descriptions of the larger context during the implementation of the intervention, procedures to recruit teams and working groups, and procedures to recruit and train process leaders.

*Reach* was calculated by dividing the number of participating teams by the total number of teams in the organization. In addition, as the approach was primarily aiming to support care teams, we identified which teams were care teams and divided the number of participating care teams by the total number of care teams in the organization.

*Dose delivered* at team level was calculated by averaging the number of actions plans by the total of participating teams. Dose delivered at working group level was calculated by dividing the amount of actual meetings planned by the maximum number of possible meetings of all participating teams.

*Dose received* at team level was an average of the percentage of team members familiar with the intervention per team. Dose received at working group level was calculated as the median extent to which goals were met per meeting (ranging from not achieved, to achieved to a very large extent), averaged over the teams. We calculated dose received for the teams that had finished all three meetings.

*Fidelity* was determined based on the process leaders’ logs by the researchers by four criteria representing the extent to which the intervention was delivered as planned for those teams that finished all three meetings. The criteria were based on the instructions of the intervention and examined whether they were adequately followed. The criteria were: “Bottlenecks and solutions were discussed in separate meetings,” “At least 2 bottlenecks were prioritized,” “At least 2 action points were prioritized,” and “Action plans were evaluated in meeting 3.″ Each criterion that was fulfilled added 25% to the score with a maximum of 100%.

*Satisfaction* at team level was an average of the mean grades per team (range 0–10) for the Healthy Working Approach, derived from workers in the intervention group that were familiar with the intervention at 6 and 9 months after baseline questionnaire. Satisfaction at working group level involved the average of the mean grades (range 0–10) per team. We calculated satisfaction for the teams that had finished all three meetings.

*Perceived positive outcomes and barriers and facilitators to the implementation* were based on interviews. These interviews were audio-recorded and transcribed verbatim. Person identifiers were removed from these transcripts. The interviews were analyzed inductively using thematic content analysis^14^ in software program ATLAS.ti (Lumivero / ATLAS.ti Scientific Software Development GmbH, https://atlasti.com/updates). Perceived positive outcomes were coded by selecting text fragments in which participants discussed positive outcomes from the intervention that they experienced themselves or observed in other individuals or entities in the organization. Barriers and facilitators to the implementation were coded by selecting text fragments in which participants discussed elements that hindered or promoted implementation of the intervention. MK and CH coded one interview separate from each other and checked whether they extracted the same data from the interview. After they had reached consensus, the remaining interviews were divided and MK and CH coded these independently of each other. Halfway through, codes were grouped into themes by CH and CB, which formed the basis for analyzing the remaining interviews by MK.

## RESULTS

Below are the results of the process evaluation of the Healthy Working Approach, implemented at the level of working groups within each team and at the level of action plans implemented in the whole team. Examples of selected problems and solutions are displayed in Text Box 1.

**Table:** 

**Examples of selected problems** Team members do not feel equally responsible for their work Working methods differ in teams, leading to confusion Team members do not trust each other Teams do not take breaks
**Examples of selected solutions** Encourage each other to provide feedback and to ask questions Working methods are renewed and included in the onboarding document for new colleagues Trust and safety will be discussed in a team meeting Start with weekly lunches

### Context

The long-term care organization underwent several internal changes during the implementation of the Healthy Working Approach (July 2021 - April 2022). First, teams moved to new buildings and small-scale living was introduced. This required major changes in the ratio of caregivers to residents and forced teams to revise their work processes. Second, multiple initiatives that touched upon healthy working were running in parallel to the Healthy Working Approach. Examples include a project on absenteeism and individual and team counseling by a newly appointed ‘sustainability officer.’ Third, the organization has practiced self-managing teams since 2015. Some teams thrived on this organizational vision, whereas others were still getting used to it or even felt an aversion to self-management. Furthermore, the implementation took place during the COVID-19 pandemic. This added to the work pressure and high sick leave and turnover rates.

*“Self-management really works for us, we collaborate nicely and the division of tasks just goes smoothly. That’s not the case for them [another team]. On all areas they were still searching.” PL 7*

*“There’re many invitations to think along. You really want the colleagues from the care teams to take the lead in thinking about certain developments and topics, doesn’t matter on what. Sometimes that was a lot.” SC 8*

### Recruitment

#### Recruitment of Teams and Working Group Members

All employees were invited to participate in this study by information and videoclips on intranet, in physical meetings and presentations, by leaflets in shared areas, and by email. The information provided an estimate of the time that was required to participate, and management supported participation (eg, by making hours available to participate). The recruitment started with individuals filling in a baseline questionnaire. When more than 30 percent of a team filled in this questionnaire, and they were willing to participate in the intervention, they could be either assigned to the intervention or control group. We chose a minimum response rate of 30% to ensure an adequate amount of data for the effect evaluation and to create support within the team for participating in the intervention. In total, 16 teams were randomized and 8 were assigned to the intervention, which we followed for this process evaluation (see Supplementary File II, http://links.lww.com/JOM/B926).

#### Recruitment and Training of Process Leaders

A role description for the process leaders was disseminated with internal communication resources in February 2021. We searched for employees who were enthusiastic about the topic of healthy working, who had a keen eye, who dared to ask open questions, who were easy to approach and knew how to create a constructive atmosphere, and who were willing to guide three teams. Eight employees were interested in fulfilling this role.

To prepare the process leaders, a train-the-trainer meeting was organized (March 2021). In the 4-hour train-the-trainer, the aspiring process leaders learnt about the Healthy Working Approach and were challenged to think critically about how to probe effectively in situations using example scenarios. They were made aware of their own role, including ensuring a safe and trusted environment, encouraging self-management, listening, summarizing and asking questions, and allowing everyone to have their say. Five employees participated in the train-the-trainer and two employees received individual training because they could not attend the train-the-trainer.

Eventually, five process leaders were willing to coach teams and were asked which teams they preferred not to be paired with in the further process, due to previous experiences or their own role in the teams. After pairing teams with process leaders, there was opportunity for process leaders to be coached 1-on-1 where needed by researcher CH. At the request of process leaders, but also to gain insight into the implementation of the meetings, CH attended four meetings. An intervision meeting for the process leaders was scheduled (December 2021), which focused on the exchange of experiences and learning from each other.

### Reach, Dose Delivered, Dose Received, Fidelity, and Satisfaction

Reach, dose delivered, dose received, fidelity, and satisfaction are displayed in Table 3. We have reached 11% of all teams and 13% of all care teams. For most participating working groups, all three meetings were delivered, and in total, 67% of all meetings were delivered. Dose delivered was 63% for the teams (ie, a plan of action was formulated) and dose received was 78% for the teams (ie, familiar with the intervention 6 and 9 months after baseline questionnaire). The meeting goals of the working groups were met to a moderate and, mostly, large extent. Fidelity was 100% in four working groups and 75% in one working group. On average, working groups graded satisfaction with the meetings a 7.6, whereas teams rated satisfaction with the approach a 6.7.

**TABLE 3:** Reach, Dose Delivered, Dose Received, Fidelity, and Satisfaction

Component	Level	Outcome
Reach: 11%	Team	11% of all teams participated
	Subgroup: care teams	13% of all care teams participated
Dose delivered: 63% team 67% working group	Team	For 63% of all participating teams was a plan of action formulated
	Working group	5 out of 8 working groups had all three meetings 1 out of 8 working groups had one meeting 2 out of 8 working groups had no meeting 16 out of 24 (67%) meetings were held
Dose received: 78% team 93% working group	Team	78% of the teams were familiar with the intervention 6 and 9 months after baseline questionnaire
	Working group	The degree to which goals were met per meeting: Meeting 1: Median of 80% of the working groups: to a large extent Median of 20% of the working groups: to a moderate extent Meeting 2: Median of 100% of the working groups: to a large extent Meeting 3: Median of 100% of the working groups: to a large extent All goals were attained to a moderate or to a very large extent. 93% of the working groups met their goals to a large extent
Fidelity: 95%	Working group	Work group 2: 100% Work group 3: 100% Work group 5: 100% Work group 6: 100% Work group 7: 75%
Satisfaction: 6.7 teams 7.6 working group	Team	6.7 (SD 0.8)
	Working group	7.6 (SD 0.6)

### Perceived Positive Outcomes

Positive outcomes of the Healthy Working Approach were reported for teams and process leaders. Participants also mentioned related positive outcomes. Below we elaborate on each category respectively.

#### Positive Outcomes for Teams

Working group members reported that the approach was helpful to address known problems and to accelerate ongoing projects. These problems and projects focused on the topics of communication and trust, collaboration, well-being, and team development. Some participants mentioned that newly adopted changes were lasting or that an ongoing development was initiated. Teams also developed a vision for the future and set corresponding goals. Second, process leaders and working group members emphasized that teams bonded. That is, workers showed more interest in each other, shared their feelings, and experienced alignment and a team identity. Next, process leaders and working groups said that the approach made workers feel heard and that these workers realized that work is not only something that can benefit others, but also themselves. Lastly, the approach led some teams to arrange training, and as a result, workers experienced professional development.

*“You feel like you’re on the same page again. And you’re super happy that you accomplish that with each other.” PL 7*

*“You just saw that they were starting to talk to each other and that they aligned and already wanted to fix some problems.” PL 4*

#### Positive Outcomes for Process Leaders

Participants indicated that process leaders experienced personal and professional development. They were triggered to step outside their comfort zone by this new role, and some applied solutions of the working groups to their own team.

*“You see other people and hear other opinions, ideas, and questions. It makes you think.” PL 1*

#### Related Positive Outcomes

Participants observed more person-centered care for residents, which was a result of actions that were set in motion by the approach. Second, participants highlighted that the approach led to the realization among employees that the health and well-being of care workers is utmost important.

*“Residents come to our meetings [of care teams], instead of going to meetings with distant therapists or not even allowed to come. So, we came closer to the residents. That’s so nice.” PL 7*

### Barriers and Facilitators to the Implementation

**FIGURE 1:**
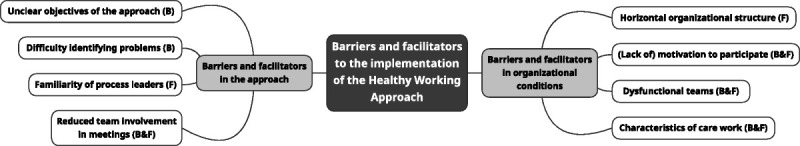
Themes of barriers and facilitators to the implementation of the Healthy Working Approach. B, barrier; B&F, barrier and facilitator; F, facilitator.

We identified barriers and facilitators to the implementation of the Healthy Working Approach rooted in the approach and in organizational conditions. Figure 1 presents an overview of the themes of barriers and facilitators.

## BARRIERS AND FACILITATORS IN THE APPROACH

Barriers and facilitators in the approach included *unclear objectives of the approach, difficulty identifying problems, familiarity of process leaders,* and *reduced team involvement in meetings.*

### Unclear Objectives of the Approach

Participants identified unclear objectives of the approach as a barrier. Workers did not know what to expect from the intervention or were under the impression that healthy working was merely about ergonomics. Consequently, it was difficult for teams to assess whether there was a need for the approach and if workers were willing to make time for it. Participants also mentioned that some workers had the misconception that the approach was mandatory, which proved demotivating to participate.

*“I of course didn’t know what to expect, so it’s difficult to know: Is there a need for this?” WM 5*

*“They didn’t actually know what they had to do. Or what awaited them. They find that scary and then they just don’t do it.” PL 3*

### Difficulty Identifying Problems

Participants considered difficulties in identifying problems in the first meeting a barrier. They reported a risk for selecting problems by working groups that were more likely consequences of a problem rather than the cause of a problem. Process leaders mentioned that working groups had difficulties with thinking outside-the-box and that some working groups selected problems that were largely outside their sphere of influence (eg, a slippery floor in the residents’ bathrooms). Additionally, participants indicated that the approach lends itself better to practical problems than to problems in the social sphere of teams.

*“It quickly became clear that that was a problem, but the problem behind the problem, that didn’t became clear.” PL 6*

*“Thinking outside-the-box, thinking about what causes it [the problem]. Yeah, I always see that as a nice process, and in this case, I think: missed opportunity.” PL 6*

### Familiarity of Process Leaders

Working group members identified familiarity of process leaders as a facilitator. They indicated that process leaders were easy to talk to and able to create a safe, informal atmosphere as a result of their familiarity with the organization. Process leaders were also reported to aid constructive meetings, because they were skilled in adding depth to conversations and keeping discussions on track. Additionally, scheduling meetings was made easier through close contact with process leaders (eg, in-person contact).

*“She [the process leader] let us think for ourselves and made us feel you could say anything.” WM 11*

### Reduced Team Involvement in Meetings

Participants identified reduced team involvement in meetings both as a barrier and facilitator. Some participants indicated that decision making in working groups was easier, whereas lengthy discussions would occur if the whole team participated in the meetings. Additionally, process leaders and working group members highlighted that working groups struggled to report back to the team, and several argued that a full team should attend the meetings to increase the effectiveness of the intervention.

*“I think if there were more of us, we would end up going on and on about things, and you would start thinking: What am I actually doing here?” WM 5*

*“It’s just a little group of a very large team. And if you see that they’re all little islands, then you may not achieve that much.” PL 7*

## BARRIERS AND FACILITATORS IN ORGANIZATIONAL CONDITIONS

Barriers and facilitators in organizational conditions included *horizontal organizational structure, (lack of) motivation to participate, dysfunctional teams,* and *characteristics of care work.*

### Horizontal Organizational Structure

Executives identified the horizontal structure of the organization as a facilitator. They reported that a horizontal structure enables teams to carry out their action plans autonomously.

*“It’s up to the teams, which fits our organization.” EX 9*

### (Lack of) motivation to participate

Participants identified motivation to participate as a facilitator and lack of motivation to participate as a barrier. Participants indicated that motivated workers prioritized healthy working and that enthusiasm of executives about the approach motivated teams to participate. However, some teams, or individuals within these teams, felt they did not need help or preferred to stay with their current routines and did not participate as a result.

*“Thinking about working conditions and what we want and where we are heading. I think that’s important, but for someone else it can be a low priority. And then it can be hard to come together.” WM 10*

*“They scheduled a vacation at the same time a meeting was planned. Then I think: well, the motivation wasn’t there in the first place.” PL 6*

### Dysfunctional Teams

Participants identified dysfunctional teams both as a barrier and facilitator. Some participants believed that dysfunctional teams were not suited for the approach. They expected these teams, for example, to get caught in complaining during the approach. Process leaders and working group members also noted that actions that touched upon the social dynamics within socially unsafe teams (eg, providing feedback) were usually not carried out, because workers were afraid of damaging their relationship with colleagues. However, other participants mentioned that dysfunctional teams acted as a facilitator, because these teams were the ones who could benefit most from the approach.

*“A team that’s really dysfunctional is not ready for this I think ... because then you get stuck in complaining very quickly and you don’t come to: What are we going to do?” WM 12*

*“Giving feedback turned out to be difficult and hard in practice, and yes, then it remained like that.” PL 6*

### Characteristics of Care Work

Participants identified characteristics of care work both as barriers and facilitators. They indicated that high and fluctuating work pressure made it hard to schedule meetings and to adhere to new agreements as priorities shifted. The topic of healthy working was said to easily lose momentum. Scheduling meetings was also hindered by irregular working hours and work schedules that were not set far in advance. Scheduling meetings was easier if a team had a resident group that did not require intensive physical care and if teams were small.

*“I don’t think it [the effect] has stuck. And that has not so much, I think, to do with the method, but more with the daily hustle and the pressure that’s on the teams.” PL 6*

*“When you provide less physical care and more guidance, you can tell residents: we have a meeting this afternoon. Are there things you want to do, and shall we do them now?” SC 8*

## DISCUSSION

Overall, the Healthy Working Approach was implemented well and, except for reach, none of the process evaluation components dropped below 60% levels. Once teams were reached, we consider levels above 60% as adequate.^6^ The implementation at working group level was generally higher compared to the implementation at team level, as well as levels of satisfaction. Furthermore, participants perceived several positive outcomes of the Healthy Working Approach for teams and process leaders. Reported outcomes for teams were faster problem solving, the development of a vision, improved team coherence, and professional development. Reported outcomes for process leaders were personal and professional development. Participants also reported related positive outcomes, including an improvement in person-centered care and increased awareness regarding the health of care workers. Furthermore, we identified barriers and facilitators to the implementation of the Healthy Working Approach that were rooted in the approach and in organizational conditions. Barriers and facilitators in the approach included *unclear objectives of the approach*, *difficulty identifying problems, familiarity of process leaders,* and *reduced team involvement in meetings.* Barriers and facilitators in organizational conditions included *horizontal organizational structure, (lack of) motivation to participate, dysfunctional teams,* and *characteristics of care work.*

### Interpretation

#### Context and Reach

The context of this approach has been challenging, as sick leave rates and work pressure were excessively high due to the COVID-19 pandemic.^15^ Additionally, the organization introduced several concurrent interventions to promote healthy working and implemented radical changes within teams as part of a relocation, such as adjustments to the ratio of caregivers to residents. Due to these contextual factors and the fact that implementation was alongside a randomized controlled trial, we reached only 11% of all teams, that were likely more eager to engage in healthy working compared to teams that were too busy to find time to participate. Teams that did manage to designate time to the approach experienced improved team coherence. This finding is in line with the randomized controlled trail that showed a positive effect of the approach on the satisfaction of the need for relatedness.^11^ A possible explanation is that the approach provided an opportunity to gather and work together as a team, after a period of severe restrictions on interactions at the start of the COVID-19 pandemic.

#### Working Group Sessions

The majority of participating teams not only managed to start a working group, but also finished all three working group meetings. Once a team signed on and a working group was set up, dose received was generally high, as well as fidelity. From the process leader log’s and interviews, it becomes clear that solutions of working groups were equally linked to both smaller problems and larger problems. This is surprising as small problems are usually chosen most often given that feasibility is one of the criteria that form the basis for the choice of a solution.^6^ Although larger problems, which involve employees outside teams, were also selected in the present study, solutions were not always found or successfully implemented. Such problems included high workload and inadequate meeting structures with physicians. More complex problems probably have a lower likelihood of adequate implementation than smaller problems. The participatory approach drives toward feasible solutions, as the likelihood of implementation is one of the criteria to prioritize solutions.^8^ Solving complex problems will likely lead to larger effects on sustainable employability, but positive effects can only be reached when the solutions are properly implemented. As a result, simple solutions for relatively smaller problems might eventually lead to bigger improvements as implementation levels are expected to be much higher.^16,17^ Although the Healthy Working Approach seems less fit to tackle team-transcending problems, the approach did prove useful for practical, smaller solutions. This tradeoff between feasibility and tackling structural problems is common to the participatory approach.^8^ Furthermore, the randomized controlled trial on the approach revealed no effect on the satisfaction of the need for competence or autonomy.^11^ Bottlenecks and solutions that could be related to autonomy or competence satisfaction were not often discussed. The process evaluation provides no insight into exactly why this is the case, but it could be that these topics are a blind spot for participants (eg, not identifying, or not knowing how to solve the issue), or that actions related to these topics needed more time to take place (eg, opting for training courses) or that actions related to relatedness are more feasible in implementation.

#### Transfer From Working Group to Team Level

Approximately 20% of the teams reported not to be familiar with the Healthy Working Approach, even though the working groups of these teams completed all three meetings. Hence, the transfer of the knowledge and action plans from the working group to the rest of the team needs more attention. However, according to organizational theory, it is difficult to create a temporary ‘intervention organizations’, like working groups, that can successfully support interventions.^18^ That is because these working groups have to meet high standards, - they need the ‘right’ workers in their group, with the ‘right’ knowledge, skills, and motivation, using the ‘right’ tools and techniques, to work together to meet the goals of the intervention.^18^ In our study, teams decided which members were part of the working groups. Given challenges like restraints in time and resources, it is likely that these working groups did not meet all these standards, which may explain why not all teams were familiar with the approach. Still, we stress the importance that teams remain in charge of who joins the working groups given that workers in self-managing teams have the best oversight on the qualities within their team. As such, we suggest that teams need more direction in how to compose a working group. Process leaders could, for example, provide information on what an ideal working group looks like. We consider an ideal working group to be versatile and diverse, because the specific problems are selected after the group is formed. In line with organizational studies on self-managing teams,^19,20^ we recommend to seek variability in age, work experience, skills, education, and personalities. Information on how to compose a working group is included in the implementation plan (Supplementary File I, http://links.lww.com/JOM/B925). An alternative is, as with other organizational-level interventions, to distribute surveys to the entire team requesting their input and use this in the working group’s meetings.^6,7^

#### Innovations in the Participatory Approach

The Healthy Working Approach relied on the participatory approach that has often been evaluated in different settings (6,7). We added innovation to the participatory approach in two ways. The present study was the first to implement the participatory approach in self-managing teams. These teams did not have an operational manager, and the responsibility of operational tasks (eg, approval of budget or scheduling workers) was carried by the teams instead. This is an important change, as the presence of an operational manager is usually a key element of the participatory approach. From a self-managing perspective, the team has the mandate to decide upon whether to participate in the Healthy Working Approach, as well as which actions to implement. However, these decisions may have not been easy for all teams, given that the level of self-management varied between teams. Participants were positive that the organization’s horizontal structure supported teams in implementing actions.

A second change to the original protocol for the participatory approach was our choice to recruit workers from the organization for the role of process leader. We intended to optimize the integration of the Healthy Working Approach in general workplace practice, which is a critical success factor for its long-term effectiveness.^21^ Our findings show that process leaders who know the organization well is an advantage that enhances opening up. According to participants, the process leaders were also well capable of realizing constructive working group meetings. Ideally, more process leaders would be available to ensure that when a future team would like to start the approach, a suitable process leader is available. Having more process leaders also increase the probability that there is a match between a certain process leader and team, meaning that both feel comfortable to take on the approach together.

### Strengths and Limitations

A strength of this study is the reflection on our strategy using a framework for process evaluations. This shows nicely which elements of the approach require additional attention. We evaluated the implementation of the intervention in eight teams, mostly care teams, but also in two teams of allied health professionals and in one team of support services. Unfortunately, we do not have sufficient data to elaborate on the implementation of the Healthy Working Approach in these latter groups. However, we found no indications in the present study that our approach was not feasible in non-care teams. Furthermore, the interviews were conducted 3–6 months after the final meeting, between June 2022 and September 2022. On the one hand, this might have affected the ability of participants to accurately recall events. On the other hand, this time interval allowed participants to report on the sustainability of the approach’s effects. Moreover, 18 working group members were invited to participate in an interview and five of them were willing to participate. Despite this limited response, the five participating working group members were part of different teams and varied in years of working experience. This leads us to expect that we have captured the most important perceived outcomes and barriers and facilitators from the perspective of working groups. Lastly, five process leaders participated in the train-the-trainer and two process leaders received individual training. It is therefore possible that the process leaders fulfilled their role differently. However, we argue that process leaders likely have their own style, regardless of training, and we have no indications that the difference in training method affected the outcomes.

### Implications for Research and Practice

Our findings demonstrate that participants perceive multiple positive outcomes of the Healthy Working Approach and we therefore recommend to implement this approach in long-term care organizations. These organizations can build upon our findings by preserving or strengthening the identified facilitators and by eliminating barriers. We also have several suggestions to improve the recruitment of teams. First, an essential condition for the implementation of changes in organizations is to obtain the support of executives.^22^ Translated to recruiting teams for the approach, executives should free up hours for teams to participate in the approach and actively stimulate teams to participate. Second, this evaluation reveals that the objectives of the approach were not entirely clear to teams and that it was therefore difficult for them to assess whether there was a need for the approach and if they were willing to make time for it. In line with Kotter’s change model,^23^ it is important in future recruitment to clearly communicate the objectives of the approach, the required time investment, and how the approach could benefit a team. This may include concrete examples and past experiences to spark motivation and thereby participation. Moreover, in this process evaluation we learned that some working groups decided to tackle problems that were outside their sphere of influence, such as a slippery floor in the bathrooms of residents. However, working groups also selected problems within their sphere of influence that proved difficult to solve, such as problems in the social dynamics of teams. We encouraged process leaders to redirect workers to other services in the organization (eg, a coach or location manager) when such issues arose. We emphasize that it is also important for teams themselves to know the route to other services in the organization, especially for self-managing teams, as they are at a large distance from managers compared to teams in hierarchical organizations. Self-managing teams that participate in the approach should thus have knowledge about the available resources in the organization. At last, we encourage researchers to study the Healthy Working Approach in long-term care organizations using realist evaluation. This methodology can help understand for which teams, under which circumstances, the approach positively affects sustainable employability. It presents an opportunity to, for example, uncover whether dysfunctional teams benefit most from the approach, and if the impact of the approach varies depending on the teams’ level of self-management. This will possibly also shed light on why a randomized controlled trial on the approach did not detect a positive effect on need for recovery.^11^ The current study did not provide an explanation for this.

## CONCLUSIONS

This process evaluation shows that, within the teams we reached, the Healthy Working Approach was implemented according to protocol, although the communication about the approach and the transfer of the action plans from the working group to teams needs attention for future implementation. An innovation of the participatory approach in the present study to implement the approach in self-managing teams proved feasible, as well as recruiting process leaders from within the organization, which was highly appreciated. Future studies on the further development of the implementation of the Healthy Working Approach in long-term care are encouraged.
